# Formononetin Treatment in Type 2 Diabetic Rats Reduces Insulin Resistance and Hyperglycemia

**DOI:** 10.3389/fphar.2018.00739

**Published:** 2018-07-18

**Authors:** Manisha J. Oza, Yogesh A. Kulkarni

**Affiliations:** ^1^Shobhaben Pratapbhai Patel School of Pharmacy & Technology Management, SVKM’s NMIMS, Mumbai, India; ^2^SVKM’s Dr. Bhanuben Nanavati College of Pharmacy, Mumbai, India

**Keywords:** type 2 diabetes mellitus, isoflavone, formononetin, SIRT1, high fat diet, streptozotocin

## Abstract

Type 2 diabetic mellitus is a multifactorial metabolic disorder affecting huge population around the world. This indicates that there is an urgent unmet need of cost effective, new treatment strategies for type 2 diabetes mellitus with no or less side effects. Phenolic compounds including isoflavones are known for their beneficial effect in metabolic disorders. The present work was intended to find out efficacy of formononetin, an isoflavone treatment in experimental model of type 2 diabetes. Type 2 diabetes mellitus was induced by feeding high fat diet for 2 weeks prior to streptozotocin administration in *Sprague Dawley* rats. Diabetic animals were treated with formononetin for 28 days at three dose level, i.e., 10, 20, and 40 mg/kg body weight orally. The effect of formononetin treatment on various parameters such as plasma glucose, glucose tolerance, insulin, HOMA-IR, lipid profile, hepatic glycogen content, glycohaemoglobin and SIRT1 expression in pancreatic tissue was measured. Histopathological changes in pancreatic tissue were also studied. Results of the study demonstrate that formononetin treatment reduces blood glucose level significantly (*p* < 0.001) at all the three dose level. It also improved glucose tolerance, insulin sensitivity and lipid profile along with reduction in glycohaemoglobin content in blood. Formononetin treatment also improved hepatic glycogen level profoundly in diabetic rats. Determination of SIRT1 expression in pancreatic tissue by immunohistochemical analysis showed that formononetin treatment increases the expression of SIRT1 in pancreatic tissue. Histopathological study showed that treatment with formononetin protects pancreatic beta cells from necro-degeneration and atrophic effect. It can be concluded that formononetin treatment reduces insulin resistance and attenuate hyperglycemia in type 2 diabetes which may be due to increasing expression of SIRT1 in pancreatic tissues.

## Introduction

Diabetes is a social catastrophe affecting more than 425 million people throughout the world, out of which 87–91% of the population is suffering from type 2 diabetes mellitus (International Diabetes Federation [IDF], 2017). Type 2 diabetes mellitus (T2D) is an outcome of combined effect of insulin resistance and insufficient insulin secretion from the pancreatic β cells (Holman et al., 2008; Axelsson et al., 2017). Uncontrolled hyperglycemia in type 2 diabetes damage vascular system, which results into vascular complications, these complications mainly affect kidney, heart, nervous system, and eyes. A recent report shows that type 2 diabetes is the leading cause of kidney failure and coronary artery diseases (Cecchini et al., 2010).

Among the several risk factors such as obesity and physical inactivity underling the prevalence of type 2 diabetes mellitus, unhealthy diet is also a major responsible factor (Al-Goblan et al., 2014). International diabetes federation as well as WHO has released healthy diet recommendation for the prevention of type 2 diabetes which includes consumption of dietary fibers in the form of legumes, vegetables, and fruit. This type of food is rich in polyphenolic compounds known for their antioxidant, anti-inflammatory, cardioprotective and antidiabetic activity (World Health Organization and UN Food, and Agriculture Organization, 2002; Mozaffarian et al., 2012; Bahadoran et al., 2013; Ley et al., 2014; International Diabetes Federation [IDF], 2015; Kulkarni et al., 2016; Oza and Kulkarni, 2016).


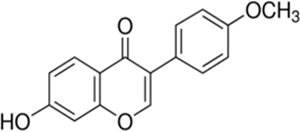


Formononetin [7-hydroxy-3-(4-methoxyphenyl)chromen-4-one] is a polyphenolic non-steroidal phytochemical with estrogenic activity (Mu et al., 2009). It is most commonly present in various legumes plants such as green peas, beans, liquorice roots, flowering tops of red clover, soy beans and Astragalus roots (Kaczmarczyk-Sedlak et al., 2013). In the clinical study, formononetin along with other isoflavone improved systemic arterial compliance (Nestel et al., 1999) and reduced arterial stiffness as well as blood pressure in postmenopausal women and obese men (Nestel et al., 2007). It provides cardioprotection by inhibiting/down regulating mitogen activated protein (MAP) kinase activity (Dubey et al., 1999). It is also a potent activator of peroxisome proliferator –activated receptors PPAR alpha and PPAR gamma which are important regulator of glucose metabolism and dyslipidemia (Shen et al., 2006). It has been reported to potentiate antihyperglycemic effect of fangchinoline through increasing insulin release from pancreatic beta cells in type 1 diabetic mice (Ma et al., 2007). In an *in vitro* study, formononetin was found to protect pancreatic beta cells by inhibiting activation of nuclear factor-kappaB (NF-κB) and reducing the production of nitric oxide which is responsible for pancreatic beta cell apoptosis in diabetes mellitus (Wang et al., 2012). Formononetin has been reported ineffective in reducing hyperglycemia in streptozotocin induced type 1 diabetes in C57BL/6 mice (Qiu et al., 2012). However, recent study report demonstrated antihyperglycemic effect of formononetin in alloxan induced type-1 diabetes in mice (Qiu et al., 2017). It has been reported to increases expression and activation of SIRT1 in *in vitro* study carried out using renal proximal tubular cell (Rasbach and Schnellmann, 2008).

Above mentioned reports show that formononetin is highly associated with the regulation of lipid and glucose metabolism by various mechanisms including SIRT1 activation. Since SIRT1, participates in regulation of glucose homeostasis through regulating hepatic glucose production, lipid metabolism and insulin production, and sensitivity, indicates its potential role to control hyperglycemia in type 2 diabetes (Shoba et al., 2009; Pulla et al., 2014). Based on these facts it has been hypothesized that formononetin may provide beneficial effect in type 2 diabetes mellitus partly by activating SIRT1. Thus the present study was aimed to find out the efficacy of formononetin to control hyperglycemia in high fat diet and low dose of streptozotocin induced type 2 diabetes in *Sprague Dawley* rats and to find out the effect of formononetin treatment in expression of SIRT1 in pancreatic tissue.

## Materials and Methods

### Chemicals

Streptozotocin was purchased from Sigma-Aldrich (St. Louis, MO, United States). Formononetin (98.8% pure) was purchased from Tokyo Chemical Industry Co., Ltd. (TCI), Japan. Insulin ELISA assay kit was purchased from RayBiotech Inc., Norcross, GA, United States. Diagnostic kits for estimation of glucose, triglyceride, cholesterol, HDL, LDL, and glycated hemoglobin were purchased from Transasia Biomedicals Ltd., India. SIRT1 antibody (B-7) was purchased from Santa Cruz biotechnology, United States. Casein was purchases from Clarion Casein Limited, Kadi, India. Cholesterol and DL-methionine were procured from Research Lab Fine Chem Industries, Mumbai, India.

### Experimental Animals

Male Sprague Dawley rats (160–170 g) were purchased from National Institute of Biosciences, Pune, India. The animals were housed in the animal facility at 22 ± 2°C, with a relative humidity of 75 ± 5% and a 12 h light/dark cycle throughout the study. Animals were fed with basal nutritional diet (Nutrimix Laboratory Animal Feed, Maharashtra, India) and provided with purified water *ad libitum*. The animals were acclimatized to the environment for 1 week before initiation of the experiment. The experimental protocol was approved by Institutional Animal Ethics Committee (Approval no-CPCSEA/IAEC/P-62/2016) of Shri Vile Parle Kelavani Mandal’s animal facility, Mumbai which is affiliated to CPCSEA (Committee for the Purpose of Control and Supervision of Experiments on Animals), Govt. of India.

The composition of regular chow was of 5% fat, 53% carbohydrate and 23% protein, with total calorific value 25 kJ/kg while the composition of high-fat diet consisting of 58% fat, 17% carbohydrate, and 25% protein as a percentage of total kcal. The high fat diet was prepared in laboratory as per the procedure described by Srinivasan et al. (2005) and Kulkarni and Garud (2016).

### Induction of Type-2 Diabetes and Treatment

Type 2 diabetes was induced using low dose of STZ (35 mg/kg, *i.p.*) after 2 weeks of dietary modification using high fat diet. The animals with plasma glucose level greater than 300 mg/dl were considered diabetic and selected for the further study (Srinivasan et al., 2005).

The suspension of formononetin and glipizide was prepared using 0.5% sodium carboxy methyl cellulose for animal feeding.

The animals in group I was receiving 0.5% sodium carboxy methyl cellulose (0.5% CMC) orally and designated as normal control animals. Group II animals were diabetic and not received any treatment throughout the study and named as diabetic control group. Diabetic animals in the groups III, IV, and V were treated with formononetin at the dose level 10, 20, and 40 mg/kg (*p.o*). Group VI animals received glipizide treatment at the dose of 5 mg/kg, orally. The treatment was given for 28 days to the animals.

### Parameters

#### Body Weight

Body weight of animals was recorded weekly.

#### Biochemical Parameters

Blood samples were collected on 0, 1, 2, 3 and at the end of 4th week collected from retro-orbital plexus using micro-haematocrit capillaries and plasma were separated by centrifugation for 15 min at 8000 rpm. Non-fasting Plasma glucose was determined using commercially available diagnostic kits as per manufacturer’s protocol (Transasia Bio-Medicals Ltd., India) in biochemical analyzer (Erba Chem-7 Biochemistry Analyzer, Germany).

At the end of the 28th day various biochemical parameters such as triglyceride (TG), total cholesterol (TC), low density lipoprotein-cholesterol (LDL-c) and high density lipoprotein-cholesterol (HDL-c) were estimated in separated plasma using commercially available diagnostic kits (Transasia Bio-Medicals Ltd., India). The estimations were performed as per the procedure mention in each kit protocol using biochemical analyzer (Erba Chem-7 Biochemistry Analyzer, Germany).

#### Glycohaemoglobin Measurement

Glycohaemoglobin was determined in freshly collected whole blood samples obtained at the end of the study using glycohaemoglobin ion exchange resin method as per manufacturer’s protocol (Transasia Bio-Medicals Ltd., India).

#### Oral Glucose Tolerance Test (OGTT)

Oral glucose tolerance test was performed at the end of 28th day. After 8 h fasting, blood sample was withdrawn by retro orbital plexus to obtain baseline blood glucose levels (0 min). Subsequently, all the rats were administered with 40% D-glucose (2 g/kg body weight) solution orally. Blood was withdrawn at 0, 30, 60, 90, and 120 min from all the groups and plasma glucose level was measured. The remaining plasma of 0 min was stored at -80°C for measurement of plasma insulin level.

#### Estimation of Plasma Insulin, Homeostatic Model Assessment-Insulin Resistance (HOMA-IR) and Insulin Sensitivity Index

Insulin level was estimated in plasma samples using rat insulin enzyme linked immunosorbent assay (ELISA) kits (RayBiotech Inc., Norcross, GA, United States). Insulin sensitivity index was calculated using the formula ISI = Ln (1/FINS^∗^FBG) and HOMA-IR were calculated using the formula HOMA-IR = (glucose × insulin)/22.5, where concentration of insulin is expressed in mIU/L and glucose in mmol/l.

#### Determination of Glycogen Level in Liver Tissue

Liver glycogen was estimated by anthron method described by Seifter et al. (1950). Briefly, 25 mg of tissue snip was boiled with 1 ml of 30% KOH for 20 min. The tissue digest was cooled and 1.25 ml of 95% ethanol was added and the content of the tube was centrifuged for 15 min at 3000 r.p.m. the supernatant was discarded and the tube was allowed to drained on tissue paper. The glycogen pellets sedimented in the tube was dissolved in 2 ml of water then treated with anthrone reagent (1:2 v/v), boiled for 10 min to develop color and optical density was measured at 620 nm within 2 h. Content of glycogen was estimated relatively from the calibration curve prepared using 02–256 μg/ml.

#### Immunohistochemistry

The sections of pancreatic tissue embedded in paraffin block was cut at 3 μm size, placed on poly-L-Lysine coated slide and incubated for 1 h. Later the sections were deparaffinized and rehydrated. The rehydrated sections were incubated in citrate buffer (pH 6) using decloaking chamber. To block the endogenous peroxidase the sections were incubated in 3% hydrogen peroxide solution for 20 min. The sections were treated with the primary monoclonal antibody, i.e., Mouse anti-rat SIRT1 (B-7) (Santa Cruz Biotechnology, Inc., United States). The visual observation was performed using diaminobenzidine color reagent and counter staining with hematoxylin. Lastly the sections were washed with Tris buffer saline (TBS) and dehydrated using alcohol. The dehydrated sections were cleared in xylene and mounted using DPX. The sections treated with antibodies were examined using light microscope to observe the intensity of antigen antibody reaction. Optical density was calculated as measure of SIRT1 expression in pancreatic tissue by ImageJ software.

#### Histopathological Examination

At the end of 28th day all the animals were sacrificed. Pancreatic tissue was dissected out and stored in 10% buffered formation solution for further study. Formalin fixed tissues of pancreas were trimmed longitudinally and routinely processed. Tissue processing was done to dehydrate in ascending grades of alcohol, clearing in xylene and embedded in paraffin wax. Paraffin wax embedded tissue blocks were sections at 3 μm thickness with the Rotary Microtome. All the slides of pancreas were stained with Hematoxylin & Eosin (H & E) stain. The prepared slides were examined under microscope to note histopathological lesions, if any.

### Statistical Analysis

All data are expressed as mean ± SEM. Results were statistically evaluated by using GraphPad Prism ver. 5.00 for Windows. Significant differences between the experimental groups were assessed by ANOVA (analysis of variance) test followed by Dunnett’s multiple comparison test. *p*-value less than 0.05 were considered to be significant.

## Results

### Body Weight

**FIGURE 1:**
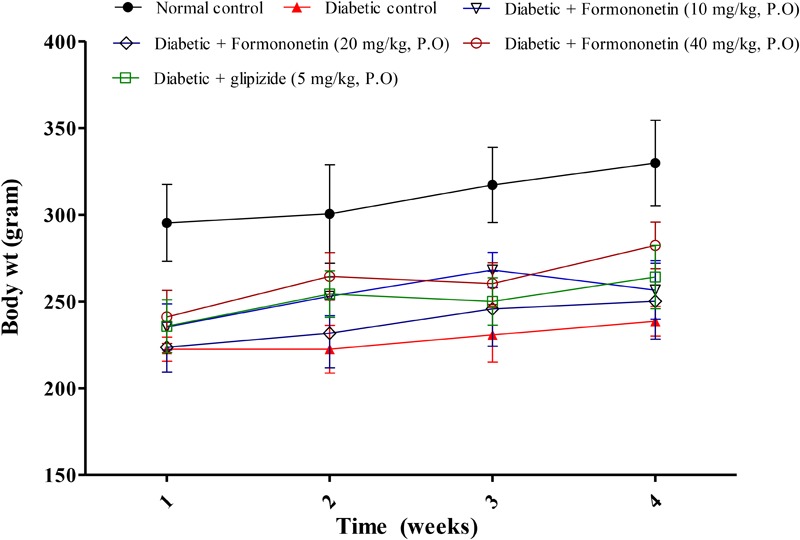
Effect of formononetin treatment on body weight in type 2 diabetic rats. Values are expressed as Mean ± SEM (*n* = 6).

Data for weekly body weight of normal, diabetic and treatment group is presented in **Figure 1**. Diabetic animals (238.7 ± 8.562) showed significant weight reduction when compared with non-diabetic animals (329.8 ± 24.65, *p* < 0.01). Treatment with formononetin improved body weight when compare to diabetic control group at selected dose level, however, the improvement in body weight was not significant.

### Effect of Formononetin Treatment on Plasma Glucose in Diabetic Rats

**FIGURE 2:**
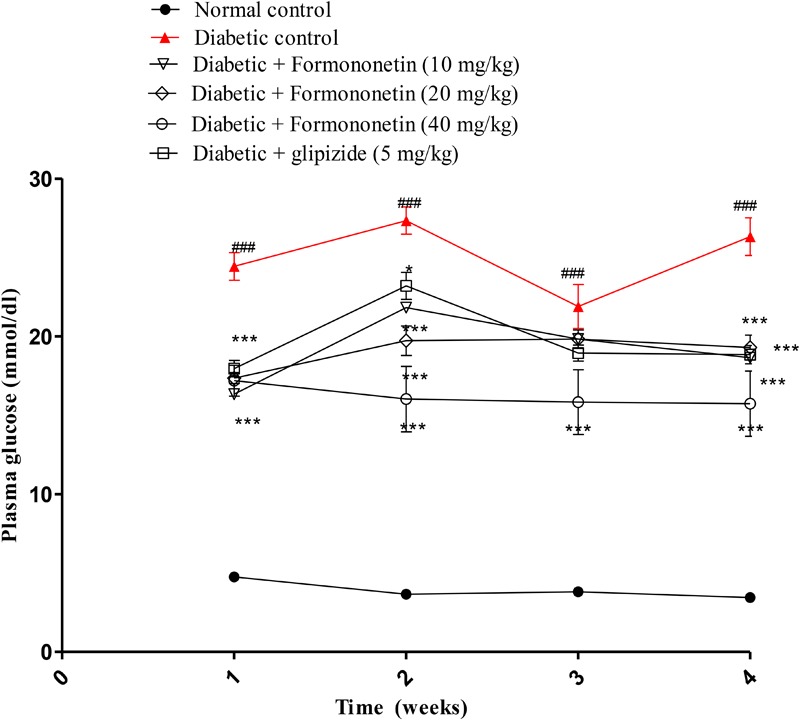
Effect of formononetin treatment in blood glucose level. Values are expressed as Mean ± S.E.M. ^∗^*p* < 0.05, ^∗∗∗^*p* < 0.001 when compared with diabetic control. ^###^*p* < 0.001 when compared to normal control (*n* = 6).

Blood plasma glucose was measured each week for all experimental animals and the results are reported in **Figure 2**. The blood glucose level in diabetic group (*P* < 0.001) was significantly higher than normal control group for each week. Interestingly, treatment with formononetin remarkably reduced blood glucose level for each week. 10 and 20 mg/kg dose of formononetin (*P* < 0.001) and standard control group animal showed significant blood glucose reduction for 1st, 2nd, and 4th week, while 40 mg/kg of formononetin dose (*P* < 0.001) showed significant reduction in blood glucose at each time point when compared with blood glucose level of diabetic animals. The blood glucose level was significantly reduced after formononetin treatment at each dose level, i.e., 10, 20 and 40 mg/kg (17.79 ± 0.9386^∗∗∗^, 19.29 ± 0.7856^∗∗∗^, and 15.73 ± 2.066^∗∗∗^) when measured at the end of 28th day treatment.

### Effect of Formononetin Treatment on Oral Glucose Tolerance

**FIGURE 3:**
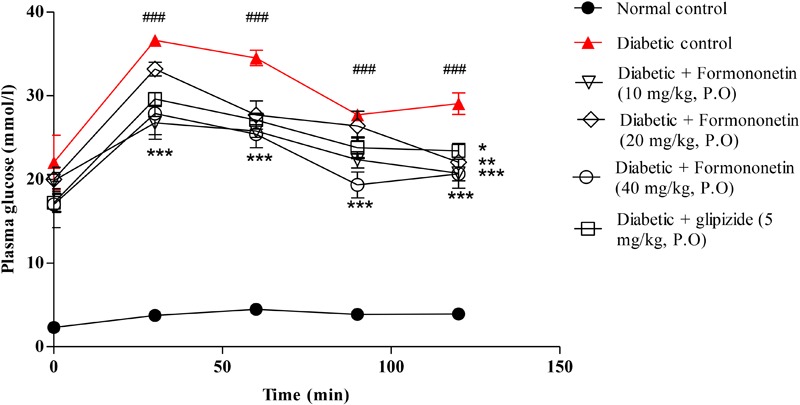
Effect of formononetin treatment on oral glucose tolerance in type 2 diabetic rats. Values are expressed as Mean ± SEM. ^∗^*p* < 0.05, ^∗∗^*p* < 0.01, and ^∗∗∗^*p* < 0.001 when compared with diabetic control. ^###^*p* < 0.001 when compared to normal control (*n* = 6).

**Figure 3** illustrated the result of oral glucose tolerance test performed at the end of the study in 8 h fasted rats. The result showed that the plasma glucose level at 30, 60, 90, and 120 min remain significantly higher in diabetic control group compared with normal control group (*p* < 0.001) and other treatment groups (*p* < 0.05, 0.01, and 0.001). The OGTT curve declined gradually at all the three dose level of formononetin treatment.

### Effect of Formononetin Treatment on Plasma Lipids in Diabetic Rats

**Table 1** shows the plasma level of triglyceride (mmol/L), total cholesterol (mmol/L), HDL-C (mmol/L), and LDL-C (mmol/L) in experimental rats of all the groups. Diabetic control group shown significant increase in TG, TC, and LDL-C (*p* < 0.001) when compared with normal control group. The plasma HDL-C level was also lowered significantly in diabetic animal (*p* < 0.001) than the normal animals. Treatment with formononetin at 10, 20, and 40 mg/kg significantly reduced triglyceride level (*p* < 0.001) in diabetic animals when compared with diabetic control group. Total cholesterol level was also reduced remarkably after treatment with formononetin at 10, 20, and 40 dose. Formononetin at lower dose level (10 mg/kg) were found in effective to improvement HDL-C level in diabetic rats, however, at higher dose level, i.e., 20 mg/kg (*p* < 0.01) and 40 mg/kg (*p* < 0.001) it shown significant increase in HDL-C level in diabetic rats. Treatment with formononetin shown significant reduction in LDL-C level at all the three dose levels (*p* < 0.01, *p* < 0.001).

**Table 1:** Effect of formononetin on level of plasma lipids in type 2 diabetic rats.

Groups	Parameters			
	TG (mmol/L)	TC (mmol/L)	HDL-C (mmol/L)	LDL-C (mmol/L)
Normal control group	0.54 ± 0.11	0.73 ± 0.07	1.32 ± 0.06	0.94 ± 0.04
Diabetic control group	3.05 ± 0.38^###^	2.87 ± 0.08^###^	0.49 ± 0.05^###^	1.97 ± 0.07^###^
Diabetic + Formononetin (10 mg/kg, orally)	1.17 ± 0.10^∗∗∗^	1.77 ± 0.60^∗^	0.93 ± 0.11	1.45 ± 0.19^∗∗^
Diabetic + Formononetin (20 mg/kg, orally)	1.04 ± 0.19^∗∗∗^	1.60 ± 0.13^∗∗^	1.08 ± 0.18^∗∗^	1.38 ± 0.061^∗∗∗^
Diabetic + Formononetin (40 mg/kg, orally)	0.63 ± 0.14^∗∗∗^	0.96 ± 0.13^∗∗∗^	1.23 ± 0.07^∗∗∗^	1.16 ± 0.11^∗∗∗^
Diabetic + glipizide (5 mg/kg, orally)	0.85 ± 0.1^∗∗∗^	1.87 ± 0.12^∗^	1.24 ± 0.18^∗∗∗^	0.99 ± 0.05^∗∗∗^

*Values are expressed as Mean ± SEM. ^∗^*p* <0.05, ^∗∗^p < 0.01, and ^∗∗∗^p < 0.001 when compared with diabetic control. ^###^p < 0.001 when compared to normal control (n = 6).*

### Effect of Formononetin Treatment on Glycohaemoglobin, Plasma Insulin and Liver Glycogen Content

As shown in **Table 2**, diabetic control group when compared with the level of glycohaemoglobin in formononetin treated group (10, 20, and 40 mg/kg) were reduced significantly at all the three dose level (*p* < 0.001). Hepatic glycogen content was significantly decreased in diabetic rats while treatment with formononetin improved hepatic glycogen level remarkably at all the three dose level, i.e., 10 mg/kg (*p* < 0.01), 20 mg/kg (*p* < 0.01), and 40 mg/kg (*p* < 0.001)^.^plasma insulin level was increased significantly after 4 weeks in diabetic rats while treatment with formononetin (*p* < 0.01) reduced plasma insulin level significantly at 10, 20, and 40 mg/kg dose. In addition formononetin treatment also improves insulin sensitivity by significantly decreasing HOMR-IR and increasing insulin sensitivity index (ISI) of the treated rats.

**Table 2A:** Effect of formononetin treatment on glycohaemoglobin and liver glycogen content.

Groups	HbA1c (%Hb)	Hepatic glycogen (mg/gm of tissue)
Normal control group	3.94 ± 0.39	281.8 ± 30.13
Diabetic control group	11.58 ± 1.54^###^	53.3 ± 8.27^###^
Diabetic + Formononetin (10 mg/kg, orally)	4.68 ± 0.21^∗∗∗^	181.7 ± 27.7^∗∗^
Diabetic + Formononetin (20 mg/kg, orally)	4.59 ± 0.46^∗∗∗^	173 ± 15.8^∗∗^
Diabetic + Formononetin (40 mg/kg, orally)	4.27 ± 0.25^∗∗∗^	271.1 ± 36.9^∗∗∗^
Diabetic + glipizide (5 mg/kg, orally)	4.56 ± 0.16^∗∗∗^	248.9 ± 30.3^∗∗∗^

*Values are expressed as Mean ± SEM. ^∗∗^p < 0.01, ^∗∗∗^p < 0.001 when compared with diabetic control. ^###^p < 0.001 when compared to normal control (n = 6).*

**Table 2B:** Effect of formononetin treatment on plasma insulin level.

Groups	Plasma Insulin (μIU/ml)	HOMA-IR	Insulin Sensitivity Index (ISI)
Normal control group	29.72 ± 3.198	3.117 ± 0.5630	(-4.168) ± 0.1796
Diabetic control group	80.37 ± 4.351^###^	84.92 ± 12.08^###^	(-7.5) ± 0.1529^###^
Diabetic + Formononetin (10 mg/kg, orally)	40.61 ± 7.12^∗∗^	36.62 ± 7.172^∗∗∗^	(-6.603) ± 0.2193^∗^
Diabetic + Formononetin (20 mg/kg, orally)	37.77 ± 9.1^∗∗^	32.68 ± 7.494^∗∗∗^	(-6.455) ± 0.2456^∗∗^
Diabetic + Formononetin (40 mg/kg, orally)	33.94 ± 6.652^∗∗^	24.68 ± 6.521^∗∗∗^	(-6.153) ± 0.2532^∗∗∗^
Diabetic + glipizide (5 mg/kg, orally)	45.92 ± 10.74^∗^	36.65 ± 10.74^∗∗∗^	(-6.562) ± 0.2285^∗^

*Values are expressed as Mean ± SEM. ^∗^*p* < 0.05, ^∗∗^p < 0.01, and ^∗∗∗^p < 0.001 when compared with diabetic control. ^###^p < 0.001 when compared to normal control (n = 6).*

### Immunohistochemistry

**FIGURE 4:**
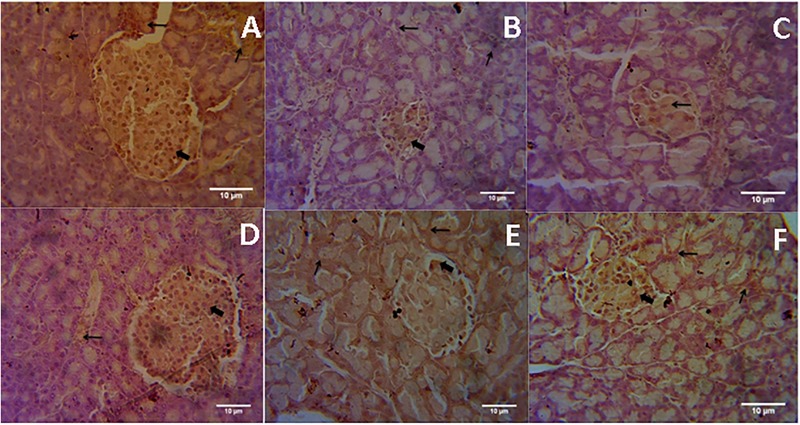
Light microscopic pictures of immunohistochemical staining. **(A)** Normal Control, **(B)** Diabetes Control, **(C)** Diabetic + Formononetin (10 mg/kg, orally), **(D).** Diabetic + Formononetin (20 mg/kg, orally), **(E)** Diabetic + Formononetin (40 mg/kg, orally), **(F)** Diabetic + glipizide (5 mg/kg, orally). Large arrow Shows expression of SIRT1 in Islet of Langerhans and small arrow shows expression of SIRT1 in acini of exocrine pancreas (SIRT1 immunostaining, 400X).

**FIGURE 5:**
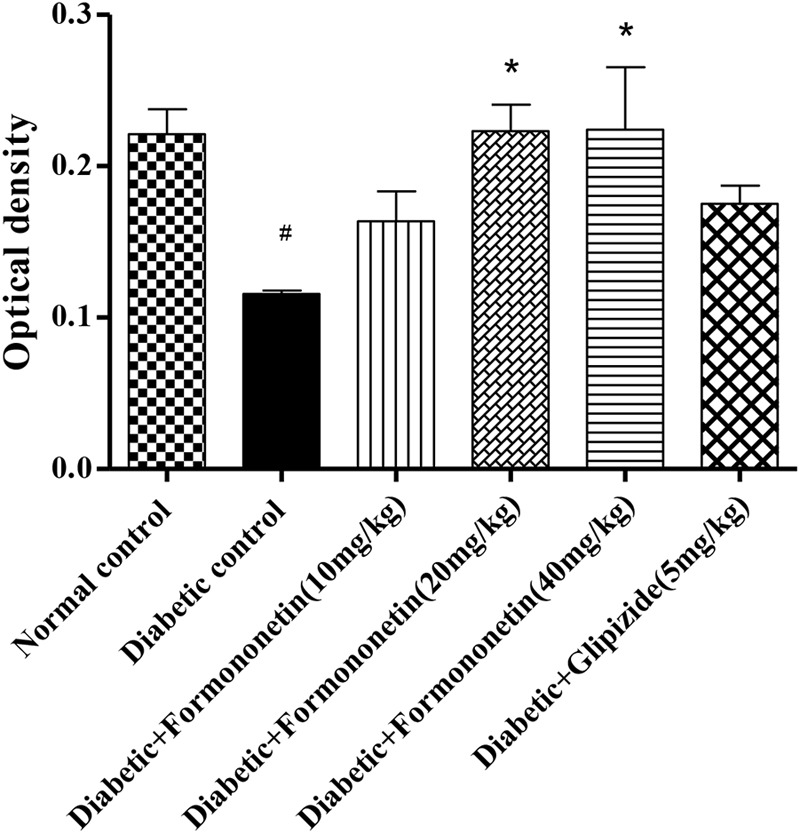
Treatment with Formononetin increases the optical density of SIRT1 in immunohistochemical staining in pancreatic tissues. All values are expressed as Mean ± SEM (*n* = 6), ^#^*p* < 0.05, when compared with normal control group, ^∗^*p* < 0.05, when compared with diabetic control group.

Microscopic examination of immunohistochemical stained slides of pancreas revealed SIRT1 expression, which was revealed by positive brown particles in the Islet of Langerhans and acini of exocrine pancreas (**Figures 4, 5**). Pancreas of normal control group showed moderate to marked SIRT1 expression in Islet of Langerhans and acini of exocrine pancreas. Expression of SIRT1 in pancreatic tissue of diabetic animals was significantly reduced when compared with normal control animals. Treatment of formononetin at the dose of 20 and 40 mg/kg increased SIRT1 expression significantly (*p* < 0.05) in pancreatic tissue when compared with diabetic animals while 10 mg/kg dose of formononetin was found to increase SIRT1 expression in pancreatic tissue when compared with diabetic control group but the difference was non-significant.

### Histopathology

**FIGURE 6:**
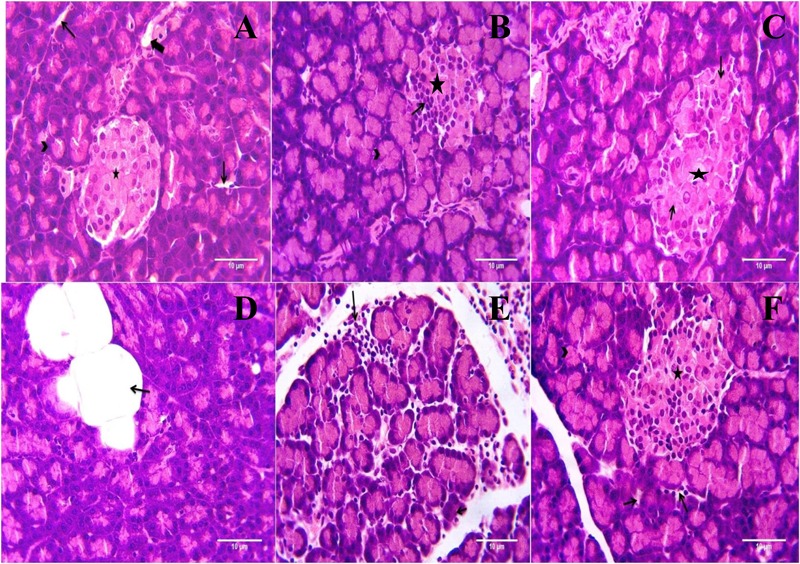
Light microscopic pictures of HE stain of pancreatic tissue. **(A)** Normal Control: Pancreas: Showing normal histology, acinus (arrow head), interlobular duct (large arrow), intercalated duct (small arrow), Islet of Langerhans (star) {H & E, 400×}. **(B)** Diabetes Control: Pancreas: Showing hyperplastic acini of exocrine pancreas (arrow head), atrophy and degeneration of Islet of Langerhans (small arrow) {H & E, 400×}. **(C)** Diabetic + Formononetin (10 mg/kg, orally): Pancreas: Showing degeneration of Islet of Langerhans (small arrow) {H & E, 400×}. **(D)** Diabetic + Formononetin (10 mg/kg, orally): Pancreas: Showing adipose tissue deposition at acini (small arrow) {H & E, 400×}. **(E)** Diabetic + Formononetin (40 mg/kg, orally): Pancreas: Showing lymphocytic infiltration (arrow) {H & E, 400×}. **(F)** Diabetic + glipizide (5 mg/kg, orally): Showing normal histology, acinus (arrow head), intercalated duct (small arrow), Islet of Langerhans (star) {H & E, 400×}.

Microscopic examination of pancreatic tissues of rats from normal control group did not revealed any lesion of pathological significance (**Figure 6**). Rats of diabetic control group and different treated groups showed various lesions in pancreatic tissue which are listed in **Table 3**.

**Table 3:** Effect of formononetin treatment on histopathology of pancreatic tissue.

Groups	Adipose deposition at acini lobule	Degeneration of islets Langerhans	Hyperplastic acini of exocrine pancreas	Lymphocytic Infiltration	Necrosis of peri-parenchymal adipose tissue	Necrosis of islets Langerhans	Atrophy of islets Langerhans
Normal control	0	0	0	0	0	0	0
Diabetic control	1	2	1	1	0	2	3
Diabetic + Formononetin (10 mg/kg, orally)	1	1	1	0	1	1	2
Diabetic + Formononetin (20 mg/kg, orally)	1	0	0	2	0	0	0
Diabetic + Formononetin (40 mg/kg, orally)	1	0	0	2	0	0	0
Diabetic + glipizide (5 mg/kg, orally)	1	0	0	0	0	0	0

*Severity of the observed lesions were recorded as 0 = Not Present, 1 = Minimal (<1%), 2 = Mild (1–25%), 3 = Moderate (26–50%), 4 = Moderately Severe (51–75%), 5 = Severe (76–100%) and distribution was recorded as focal, multifocal and diffuse.*

## Discussion

Polyphenolic phytochemicals such as resveratrol, catechin, and genistein are known for their antidiabetic effects (Tsuneki et al., 2004; Cao et al., 2007). Isoflavone, a type of polyphenolic compounds has been reported to increase insulin secretion and improves glycemic control in diabetics along with antioxidant potential (Rüfer and Kulling, 2006; Lu et al., 2008). Hyperlipidemia, Oxidative stress and increase in inflammatory processes are responsible for the pathogenesis of type 2 diabetes (Butkowski and Jelinek, 2017). Formononetin has been reported for its hypolipidemic, anti-inflammatory and antioxidant activity. It also activates PPAR receptors and inhibits the activation of NF-kβ. Formononetin also showed inhibitory effect on beta cell apoptosis and also induced beta cell regeneration (Shen et al., 2006; Qiu et al., 2017). Formononetin also reported to increase the expression of SIRT1 class III histone deacetylase which is associated with regulation of glucose metabolism. Thus the study was planned to evaluate the efficacy of formononetin in type 2 diabetes mellitus which is the most prevalent type of diabetes.

There are various animals models established to mimic type 2 diabetic conditions in animals. In the present study, *Sprague Dawley* rats were chosen as experimental animals to obtain pathological features similar to type 2 diabetes mellitus in human (Båvenholm et al., 2001). Reduction in insulin sensitivity and pancreatic beta cell dysfunction are the two key factors responsible for the pathogenesis of type 2 diabetes. It is well proven that diet rich in high fat content is one of the responsible factors for the development of insulin resistance (Ohtsubo et al., 2011). Streptozotocin is used to induce type 1 as well as type 2 diabetes. It has been reported that the severity of beta cell destruction depends on dose of STZ, so low dose of STZ is used to induce type 2 diabetes (Zhang et al., 2003; Srinivasan et al., 2005). Thus, to develop insulin resistance and partial β cell dysfunction in the experimental animals high fat diet was introduced along with low dose of streptozotocin to mimic type 2 diabetic conditions in experimental rats.

Diet modification for 2 weeks with high fat diet before administration of streptozotocin to induce insulin resistance syndrome in rats is universally accepted and well-studied disease model for development of type 2 diabetic conditions in experimental animals (Srinivasan et al., 2005; Gheibi et al., 2017). So in the present study, all the animals except normal control animals were fed with high fat diet for 2 weeks before STZ administration to produce insulin resistance. High fat diet must contain 40–60% of fat composition to generate insulin resistance thus the high fat diet used in the study was containing 58% fat, 25% protein and 17% of carbohydrate. Low dose below 45 mg/kg of streptozotocin is recommended by various authors to induce type 2 diabetic conditions in rodent so in the present study the selected dose of streptozotocin was 35 mg/kg of body weight (Zhang et al., 2003; Srinivasan et al., 2005).

Body weight in diabetic animals was significantly reduced when compared with normal animals while treatment with formononetin improved the body weight which may be due to better glycemic control.

The results showed that high fat diet and low dose of streptozotocin induced hyperglycemia and hyperinsulinemia in experimental animals, and treatment with formononetin showed significant reduction in plasma glucose level. Results obtained from oral glucose tolerance test also support these findings. Formononetin treatment at 40 mg/kg dose level showed maximum improvement in oral glucose tolerance compare to diabetic control group animals. Formononetin treatment also reduced plasma insulin concentration significantly in diabetic rats. This indicates its role in improvement of insulin sensitivity. In addition treatment with formononetin enhanced insulin sensitivity index and reduced HOMA-IR significantly at all dose level indicates improved glucose metabolism and proper utilization of insulin by tissues and reduction in insulin resistance at tissue level. Glucose homeostasis in the body is mainly depends on proper insulin secretion from pancreatic beta cells and tissue sensitivity toward insulin to increase glucose uptake. In normal condition insulin and hyperglycemia promotes glucose disposal through suppression of hepatic glucose production, stimulation of glucose uptake by liver and peripheral tissues. However, in type 2 diabetic condition these mechanisms are disturbed results into insulin resistance and hyperglycemia (Shanik et al., 2008; Cersosimo et al., 2015). In response to hyperglycemia and insulin resistance, insulin secretion is increased by pancreatic beta cells to maintain normal glucose homeostasis leads to hyperinsulinemia in the initial stage of type 2 diabetes. Hyperinsulinemia further increases insulin resistance by damaging insulin receptor at cell site including pancreatic beta cells and responsible for reduction in insulin secretion in later stage of type 2 diabetes (Kulkarni et al., 1999). In type 2 diabetic patients due to hyperglycemic condition glucose uptake by hepatic tissue is impaired result into glucose intolerance in type 2 diabetic patients (Mari et al., 1994).

Protein glycation is natural process including formation of glycohaemoglobin in which glucose reaction take place with hemoglobin at N-terminal end of β-chain and results into formation of glycohaemoglobin (HbA1c). Our result shows that formononetin is able to control blood glucose level and thus the formation of glycohaemoglobin. It is well known that the normal limit for glycohaemoglobin is 3–6%, however, in type 2 diabetes it will increase up to 6–12% due to persistent hyperglycemia. It is the best indicator of a blood glucose concentration for the previous 4–6 weeks in individual not just at the time of measurement. It is well documented that strict control in blood glucose can decline the rate of formation of glycohaemoglobin because of the linear correlation between blood glucose level and formation of glycohaemoglobin (Koenig et al., 1976; Sherwani et al., 2016). Our finding represents that formononetin treatment to the diabetic rats significantly reduced the formation of glycohaemoglobin and hyperglycemia in dose dependent manner when compared with non-treated diabetic animals.

It is well documented that hepatic glucose production in postabsorptive state is mainly due to glycogenolysis and gluconeogenesis (Cherrington, 1999) insulin resistance at hepatic cells leads to increased hepatic glucose output via glycogenolysis results into decrease in hepatic glycogen concentration. In our study, liver glycogen level was profoundly reduced in diabetic animals which may be due to over glycogenolysis in hepatic cells. However, treatment with formononetin at all dose level remarkably improved hepatic glycogen level, this indicates that formononetin may have role to inhibit hepatic glucose production. Similar findings has also been reported in literature for metformin, which controls hyperglycemia partly by reducing hepatic glucose output. The reduction in 50% of hepatic glucose output by metformin is mainly due to inhibition of glycogenolysis (Cusi et al., 1996).

Insulin resistance play important role in development of dyslipidemia in type 2 diabetes. It alters triglyceride rich lipoprotein metabolism and increase hepatic lipase activity results into increased level of triglyceride, cholesterol and LDL with reduction in HDL cholesterol in diabetics (Krauss, 2004). Plasma lipid profile in diabetic animals was impaired significantly when compared with normal animals, however, treatment with formononetin improved lipid profile significantly in dose dependent manner. It has been observed that 10 mg/kg dose is not effective in improving HDL-C concentration significantly in diabetic animals. Our results are also supported by other study conducted using formononetin which showed that formononetin effectively provide hypolipidemic in albino rats. Furthermore formononetin is also reported as potent activator of receptors PPAR-α and PPAR-γ which is associated with regulation of lipid metabolism (Sharma, 1979; Shen et al., 2006).

Histopathological examination of the pancreatic tissue revealed presence of inflammatory, degenerative, necrotic, hyperplastic lesions in the endocrine as well as exocrine part of the pancreas in diabetic animals. Atrophy of islets of Langerhans has also been observed in diabetic control animals. Treatment with formononetin at the dose of 10 mg/kg showed little improvement in histological damage caused due to diabetic condition, however, higher dose of formononetin showed only lymphocytic infiltration and absence of necrosis and atrophy of islet of Langerhans. It has been observed that chronic feeding of high fat diet till the end of the study showed deposition of excessive fat in acinar lobules of pancreas in diabetic as well as formononetin and glipizide treated animals. This indicates that formononetin treatment has protective effect on pancreatic tissue damage.

SIRT1 is an NAD dependent class III histone deacetylase participates in regulation of glucose homeostasis through regulating hepatic glucose production, lipid metabolism and insulin production, and sensitivity (Shoba et al., 2009; Pulla et al., 2014). It also decreases hepatic glucose production via deacetylation and activation of the AMPK kinase LKB1 (Clark-Knowles et al., 2014; Yacoub et al., 2014). It regulates glycolysis, gluconeogenesis and fatty acid β-oxidation by activating PGC-1α transcription coactivator and stimulating insulin secretion. In pancreatic β cells SIRT1 controls activity of various transcription factors and co-regulators like p300, p53, Ku70, UCP2, NF-kβ, HNF4α, FOXO, PPARγ and regulates insulin secretion as well as protects β-cells from oxidative stress (Shoba et al., 2009; Vassilopoulos et al., 2011). It also regulates lipid mobilization in adipose tissue and glucose uptake by skeletal muscles (Kitada and Koya, 2013).

Pancreatic SIRT1 immunohistochemical study result showed that in diabetic rats SIRT1 expression was intensely reduced when compare with non-diabetic animals. While treatment with formononetin significantly improved SIRT1 expression in pancreatic tissue at the dose of 20 and 40 mg/kg.

## Conclusion

The present study shows that formononetin possess antidiabetic effect in high fat diet and low dose streptozotocin induced type 2 diabetes in experimental rats which may be linked with increase in expression of SIRT1. However, detail study is required to find out the molecular pathways involved in antihyperglycemic effect of formononetin via increasing SIRT1 expression in pancreatic tissue.

## Author Contributions

All authors listed have made a substantial, direct and intellectual contribution to the work, and approved it for publication.

## Conflict of Interest Statement

The authors declare that the research was conducted in the absence of any commercial or financial relationships that could be construed as a potential conflict of interest. The reviewer MB-Z and handling Editor declared their shared affiliation.
